# The Effect of Polyphenol Supplementation in People with Multiple Sclerosis: A Systematic Review of Clinical Trials

**DOI:** 10.3390/nu18121875

**Published:** 2026-06-10

**Authors:** Lauren Brooks, Aqif Farhan bin Azmil Farid, Amudha Poobalan, Alexandra Johnstone, Phyo Kyaw Myint

**Affiliations:** 1 School of Medicine, Medical Sciences and Nutrition, University of Aberdeen, Aberdeen AB25 2ZD, UK; 2 North Bristol NHS Trust, Southmead Hospital, Bristol BS10 5NB, UK; 3 Aberdeen Royal Infirmary, Aberdeen AB25 2ZN, UK; aqiffarhan.azmilfarid2@nhs.scot; 4 The Institute of Applied Health Sciences, School of Medicine, Medical Sciences and Nutrition, Foresterhill Campus, University of Aberdeen, Aberdeen AB25 2ZD, UK; a.poobalan@abdn.ac.uk; 5 The Rowett Institute, School of Medicine, Medical Sciences and Nutrition, University of Aberdeen, Foresterhill Health Campus, Ashgrove Road West, Aberdeen AB25 2ZD, UK; alex.johnstone@abdn.ac.uk

**Keywords:** multiple sclerosis, polyphenols, curcumin, flavanols, phenolic acids, cognition, fatigue, depression, dietary supplements, inflammation, systematic review

## Abstract

**Background/Objectives:** Multiple sclerosis (MS) is an autoimmune neuroinflammatory disease affecting 2.9 million worldwide. Current immunosuppressive treatments offer limited neuroprotection and often cause adverse effects. Polyphenols with antioxidant and anti-inflammatory properties have been investigated as adjuncts in MS. **Methods**: On 19 June 2025, Embase, Medline, and ClinicalTrials.gov were systematically searched. Eligible clinical trials assessing polyphenol supplementation in MS were included. Outcomes of interest were Expanded Disability Status Scale (EDSS), annualised relapse rate (ARR), magnetic resonance imaging (MRI) changes, safety, and tolerability. Risk of bias was evaluated using the Cochrane tool, and certainty of evidence was appraised with Grading of Recommendations Assessment, Development, and Evaluation (GRADE). The protocol was registered with PROSPERO (CRD420251052042). **Results**: Of 870 records identified, 13 trials (*n* = 785) met inclusion. Nanocurcumin consistently improved EDSS in relapsing–remitting MS (3 trials, *n* = 150; *p* = 0.039–0.041), while epigallocatechin-3-gallate, silymarin (SM), cranberry extract, and bio-enhanced curcumin extract (BCM-95) curcumin showed no significant impact on disability, relapse rates, or MRI outcomes. Intervention adverse events were generally mild. SM showed potential hepatoprotective effects. Risk of bias was determined as low risk for seven of the trials and of some concern for five of the studies. Most often raising concerns because of selective reporting. Certainty of evidence, assessed using GRADE, was generally moderate, indicating some uncertainty regarding the outcomes. Meta-analysis was not possible due to the heterogeneity of included studies. **Conclusions**: Nanocurcumin may contribute to improvements in disability outcomes in Relapse Remitting Multiple Sclerosis (RRMS), whereas other polyphenols lack consistent efficacy. However, the evidence base remains limited by small sample sizes and methodological concerns. Larger, multicentre randomised controlled trials are required to establish optimal dosing, long-term safety, and therapeutic potential.

## 1. Introduction

As of 2024, it is estimated that multiple sclerosis (MS) affects 2.9 million people worldwide, and the number of those affected in the UK is growing by 2.4% annually. MS causes significant disabling symptoms leading to premature death, with life expectancy reduced by 2 to 7 years [1]. MS typically presents in females under 40 with optic neuritis, transverse myelitis, fatigue, motor dysfunction, and cognitive impairment. Mean age at diagnosis is 30 years old, with most being diagnosed between the ages of 20 and 50, and it is twice as common in females [2]. The subtypes relapse–remitting multiple sclerosis (RRMS), primary progressive multiple sclerosis (PPMS), and secondary progressive multiple sclerosis (SPMS) are defined by clinical course. Although MS has traditionally been considered an autoimmune disease driven by autoreactive T-lymphocytes, its pathophysiology is increasingly recognised as complex and multifactorial. Both adaptive and innate immune mechanisms contribute to disease initiation and progression, involving T-cells, B-cells, microglia, and peripheral immune cell infiltration across a disrupted blood–brain barrier [3]. Dysregulation of immune pathways, including activation of nuclear factor kappa B (NF-κB) signalling and imbalance between pro-inflammatory Th17 cells and regulatory T-cells (Tregs), promotes chronic neuroinflammation and neuronal injury. Increased oxidative stress and reactive oxygen species production further contribute to demyelination, axonal damage, mitochondrial dysfunction, and irreversible neurodegeneration [4]. BBB disruption also facilitates ongoing leukocyte migration into the central nervous system, amplifying inflammatory cascades and disease progression. MS is diagnosed according to the McDonald criteria, whereby there are neuroradiological changes seen on T2/gadolinium MRI scans along with the presence of oligoclonal bands in cerebrospinal fluid (dissemination in space) [5]. These changes should be present on more than one occasion (dissemination in time) for a formal diagnosis to be made [6]. Treatment focuses on symptom control, managing acute attacks, and slowing disease progression. No current therapies promote remyelination or neuronal repair, although several are under study. There are now over 20 approved disease-modifying drugs [7] that work by immunosuppression, carrying risks of infection susceptibility alongside risks of allergic reactions, arrhythmias, liver enzyme elevation, headaches, and flu-like symptoms. These often lead to early withdrawal from treatment [8]. Financial barriers and health inequalities can further limit treatment in some settings. Polyphenols are naturally occurring plant compounds characterised by multiple phenolic structures and are known for their strong antioxidant and anti-inflammatory properties [9]. Experimental evidence suggests that polyphenols may modulate several pathological mechanisms relevant to MS, including inhibition of NF-κB activation, reduction in pro-inflammatory cytokine production, restoration of Treg/Th17 immune balance, attenuation of oxidative stress, and preservation of BBB integrity. Through these mechanisms, polyphenols may reduce neuroinflammation and limit demyelination and neuronal injury. Existing evidence investigating polyphenol supplementation in neurodegenerative and inflammatory diseases has shown mixed but generally promising outcomes. However, previous reviews in the MS field have often focused on single polyphenol compounds, included heterogeneous study populations and interventions, or lacked detailed evaluation of mechanistic pathways and clinical outcomes, limiting the ability to draw broader conclusions regarding the therapeutic role of polyphenols in MS.

Attention deficit hyperactivity disorder (ADHD), much like MS, involves immune dysregulation and oxidative stress. ADHD has been associated with increased oxidative stress and neuroinflammation, including imbalances between oxidants and antioxidants along with immune dysregulation. Additionally, ADHD has been linked with inflammatory processes, altered cytokine profiles, and associations with immune-related conditions, supporting the role of immune mechanisms in its pathophysiology [10]. A recent 2024 systematic review of ten studies (*n* = 556) found that the polyphenol epigallocatechin-3-gallate (EGCG) improved hyperactivity and impulsivity seen in individuals with ADHD. Higher dietary polyphenol intake is linked to reduced ADHD risk. Polyphenol supplementation was shown to rebalance oxidative stress pathways and reduce neuroinflammation, as evidenced by lower oxidised glutathione levels. Taken together, these shared oxidative stress and immune-mediated pathways provide biological plausibility for the therapeutic potential of polyphenols in MS and support the rationale that these compounds may modulate redox balance and neuroinflammation [11].

A 2021 systematic review evaluating the impact of polyphenol supplementation on cognitive function identified improvements in verbal memory and executive function correlating to reduced IL-6 and TNF-a. Proposed mechanisms include antioxidant activity, NF-kB pathway inhibition and improved endothelial function [12]. Given these findings and overlapping pathophysiology and symptomology in MS and cognitive decline, polyphenols may offer clinical benefit in MS.

On initial search, a limited systematic review examining EGCG supplementation in MS reported reductions in the pro-inflammatory cytokine IL-6, alongside lower anxiety levels and improved quality of life. However, this review focussed exclusively on EGCG as a single polyphenol compound with limited intra-group analysis and comparisons between intervention and placebo groups. This limits the generalisability of findings to other polyphenols or broader polyphenol-rich interventions [13].

A more comprehensive review comparing different polyphenols to understand their strengths and weaknesses with regard to clinical manifestations on MS is needed.

## 2. Materials and Methods

An open-ended, language-restricted (English) search was performed on 19th June 2025 to screen for registered trials on the effect of polyphenol supplementation in humans with MS. Embase 1974 to 2025 and Medline ^®^, both via OVID, along with clinicaltrials.gov were screened by one reviewer (LM) in addition to a manual snowball search. This ensured inclusion of all eligible studies with the following criteria: Population: Adults (>18 years) with a diagnosis of multiple sclerosis of any subtype. Studies on animals, in vitro/ex vivo models of MS or published in languages other than English were not eligible. Intervention: Isolated polyphenol supplementation of any duration and dose, excluding those using polyphenol combinations or non-polyphenol intervention. Comparator: placebo-controlled only. Outcome: All reported outcomes were considered, with no restrictions on study design provided that primary data were collected. Outcomes analysed included Expanded Disability Status Scale (EDSS), which is a way of measuring disability and progression of MS; annualised relapse rate (ARR), which estimates the number of relapses a patient experiences each year; and MRI changes, safety, and tolerability, with particular attention to effects on liver function tests (LFTs). The protocol was registered with PROSPERO (CRD420251052042). This systematic review was conducted and reported in accordance with the PRISMA 2020 Statement [14].

The primary MeSH term ‘polyphenol’ was used along with exploring related terms and the scope note. Narrower subclasses and related terms were then identified to capture all polyphenols. Free-text terms were combined with MeSH terms using Boolean operators.

### Data Extraction and Synthesis

Data extraction was conducted using a standardised, pre-piloted excel form. All data were extracted by a single reviewer (LB). For each eligible study, information was collected on key study characteristics (Table 1) and study interventions (Table 2). Outcome data were extracted for the following domains: EDSS, ARR, MRI changes, safety, and tolerability. Effect estimates (*p*-values) and measures of variability (e.g., standard deviations, confidence intervals) were extracted when reported. Insufficient data were available to derive effect sizes.

Extracted data were organised into tables to facilitate cross-study comparison, including a dedicated table summarising intervention characteristics. A second reviewer (AFAF) independently verified the data extraction to ensure accuracy and consistency. Discrepancies were discussed and resolved through consensus. Throughout the extraction process, decisions were made in accordance with the predefined protocol.

Risk of bias was assessed (by LB) using the Cochrane risk of bias tool V1, grading as low, some concern, high, or unclear risk of bias [15]. Certainty of evidence was evaluated using GRADE [16]. No ethical approval was required due to the absence of primary data collection [17]. Outcomes were manually extracted into tables for comparison, though not all trials reported the same measures.

**Table 1:** Patient demographics and study design for 13 included studies. Sample sizes ranged from 10 to 122 and participant ages between 23 and 59 years. As expected, given the demographics of MS, most participants were female (68%). Most of the studies were RCTs. Studies were performed in a variety of countries, most commonly Iran and Germany.

Reference	Participants	Age	Gender (F = Female) (M = Male)		Trial	Year	Country	Subtype of MS
			Intervention	Placebo				
Louvera et al., 2015 [18]	Ph1: 10. Ph2: 13 total	Ph1: 39–56 Ph2: 33–59	F:8 M:1	F:19 M:3	Pilot study. Ph1: open label, Ph2-RCT	Ph1: Sep 2009–April 2012. Ph2: July 2011	US	RRMS or SPMS
Mähler et al., 2015 [19]	18	32–52	F:10 M:8	n/a	RCT (crossover)	2011–2012	Germany	Any
Rust et al., 2021 [20]	38	41–58	F:14 M:16	F:13 M:17	RCT	2009–2016	Germany	PPMS/SPMS
Petracca et al., 2021 [21]	80	26–45	F:12 M:28	40 total	RCT	April 2012–April 2014	Italy	Any
Ghiasian et al., 2021 [22]	48	23–47	F:22 M:5	F:24 M:3	RCT	2019–2020	Iran	Any
Bellmann-Strobl et al., 2021 [23]	122	29–50	F:41 M:21	F:40 M:20	RCT	2007–2009	Germany	Any
Dolati et al., 2018 [24]	41	28–51	F:13 M:7	F:13 M:9	RCT	June 2016–Jan 2017	Iran	Any
Dolati et al., 2019 [25]	50	29–40	F:16 M:9	F:16 M:10	RCT	Aug 2021–Sep 2022	Iran	RRMS
Dolati et al., 2018 [26]	50	28–51	F:16 M:9	F:15 M:10	RCT	2017	Iran	RRMS
			Plus, healthy controls (F:22, M:13)					
Gallien et al., 2014 [27]	171	39–59	F:63 M:19	F:62 M:27	RCT	Jan 2006 to Nov 2007	France	Any
Abbasirad et al., 2021 [28]	54	27–51	F:22 M:5	F:24 M:3	RCT	2020	Iran	RRMS
Coe et al., 2019 [29]	40	30–52	F:14 M:5	F:16 M:5	Feasibility trial	May 2016–Aug 2017	UK	RRMS
Dolati et al., 2018 [30]	50	26–51	F:42 M:8	F:22 M:13	RCT	June 2016–Jan 2017	Iran	RRMS

**Table 2:** Detailed overview of the interventions included epigallocatechin-3-gallate (EGCG), nanocurcumin, BCM-95 curcumin, cranberry extract, SM, and flavanol formulations within trials and their durations. All interventions were orally administered.

Reference	Intervention	Concomitant Treatment
Louvera et al., 2015 [18]	400 mg EGCG BD for 6 months of PH1, 12 months for PH2 + F2 + I3	Stable on glatiramer acetate for 6 months prior to study
Mähler et al., 2015 [19]	600 mg EGCG (split into BD 300 mg—150 mg capsules) or placebo for 12 weeks, then wash out for 4 weeks then swap. Placebo was starch.	Stable on Glatiramer.
Rust et al., 2021 [20]	100 mg BD EGCG for 3 months, 400 mg OD for 3 months, 600 mg OD for 12 months, 800 mg OD for 12 months, 1200 mg OD for 6 months. Those who agreed to treatment continuation for a further 12 months had 200 mg OD then escalated by 200 mg every 2 weeks reaching 1200 mg after 10 weeks.	Standard MS therapies were allowed if stable.
Petracca et al., 2021 [21]	BCM 95-micronised curcumin with turmeric essential oils 500 mg orally for 24 months	Continuation of any existing MS therapies.
Ghiasian et al., 2021 [22]	140 mg Silymarin OD for 6 months	Fingolimod (FTY720) once a day (an existing MS therapy).
Bellmann-Strobl et al., 2021 [23]	800 mg capsule of sunphenon (GTE containing >90% EGCG) or identical placebo for 18 months.	Continuation of any existing MS therapies.
Dolati et al., 2018 [24]	80 mg/day nanocurcumin capsules for 6 months	Continuation of any existing MS therapies.
Dolati et al., 2019 [25]	80 mg/day nanocurcumin for 6 months.	Continuation of any existing MS therapies.
Dolati et al., 2018 [26]	80 mg OD nanocurcumin—6 months.	Continuation of any existing MS therapies.
Gallien et al., 2014 [27]	Oral cranberry extract with 18 mg proanthocyanidins BD, 12 months	Continuation of any existing MS therapies.
Abbasirad et al., 2021 [28]	SM (420 mg in three divided doses) for 6 months.	Continuation of any existing MS therapies.
Coe et al., 2019 [29]	Low flavanol (0.99 mg/g of catechins) vs. high (8 mg/g catechins) for 6 weeks	Continue existing first line MS therapies including glatiramer acetate, interferone beta, teriflunomide and dimethyl furmarate.
Dolati et al., 2018 [30]	80 mg OD nanocurcumin—6 months.	Weekly interferon beta-1a (Actovex) (Gemabiotech S A, Sante Fe, Argentina) injections for at least 3 months before the intervention and they were weekly under Actovex treatment during supplementation.

## 3. Results

**Figure 1:**
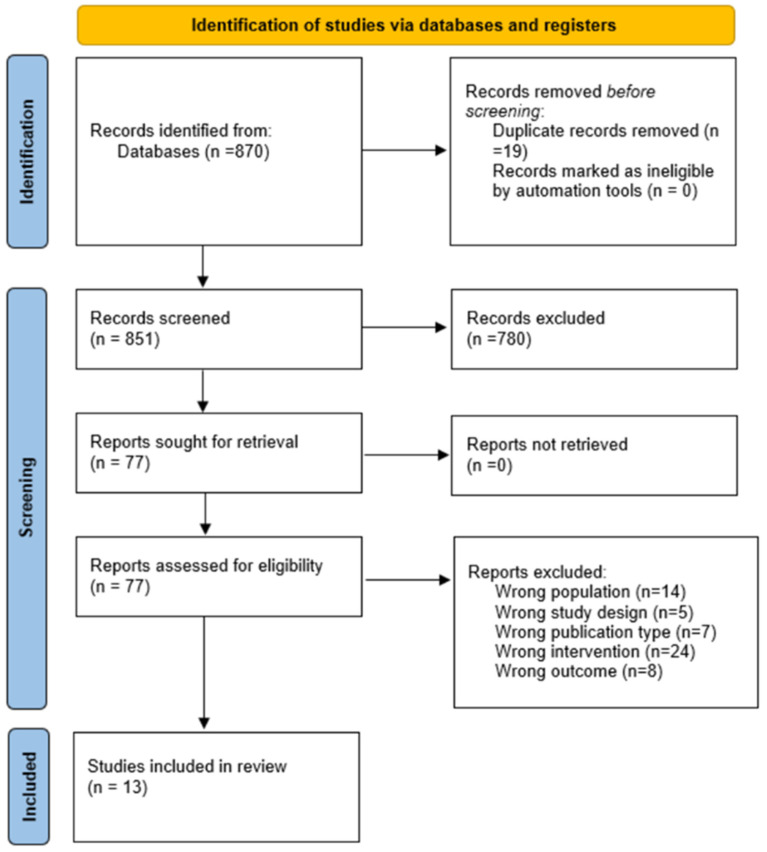
PRISMA flow diagram illustrating the study selection process including reasons for exclusion at full-text screening stage [32].

Initial database screening retrieved 870 records. These were imported into Rayyan [31] for automatic duplicate removal. Screening was conducted by a single reviewer, beginning with titles and abstracts, followed by full-text assessment. Initial exclusion due to many studies being conducted in vitro/ in vivo with interventions which did not meet inclusion criteria After this process, 13 studies (*n* = 785) met the eligibility criteria. This process is demonstrated in Figure 1 [32]. Sample sizes ranged from 10 to 122, and participants aged between 23 and 59 years. As expected, given the demographics of MS, most participants were female (68%) [33]. Participant characteristics are summarised in Table 1 and Table 2.

**Figure 2:**
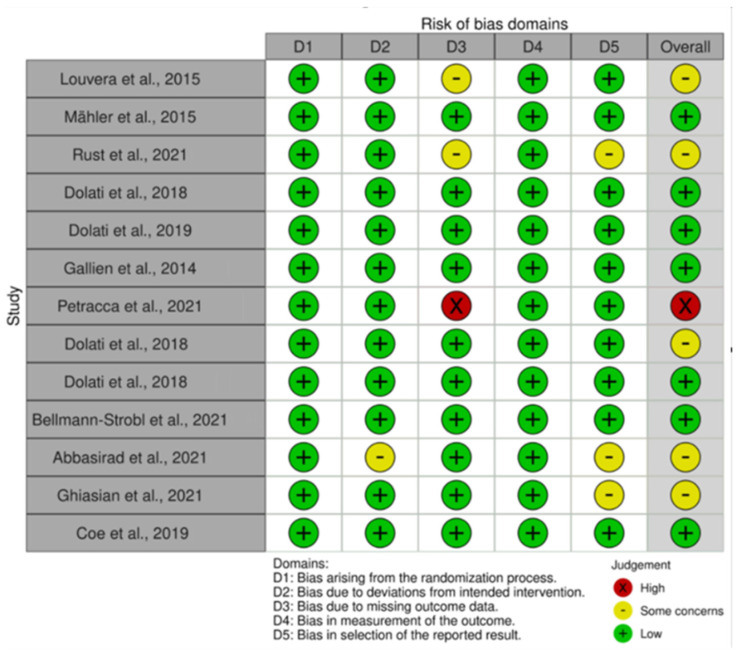
Risk of bias assessment using Cochrane Risk of Bias 2.0 (Rob 2) [11] tool across 5 domains. Visualised using the Rovis tool [18,19,20,21,22,23,24,25,26,27,28,29,30,34].

This search process, which applied minimal exclusion criteria, was designed to comprehensively identify all eligible studies and ensure a thorough review of the available evidence in relation to the stated objectives. The search strategy was limited by date range (reflecting recent practice) and language (restricted translation service availability). Of the thirteen studies, five had low risk of bias, and seven raised some concern, most frequently due to selective reporting, with several studies presenting only a single outcome measure or time point. Additional concerns were from missing outcome data due to early trial withdrawal. In many cases, it was unclear if these data were included in the final analysis, as intention-to-treat (ITT) principles were not consistently applied. This is visualised in Figure 2.

Regarding the quality of evidence assessment using GRADE, Louvera et al. (2015) [18] was rated as very low. This was due to inconsistency between Phase 1 and Phase 2 results, along with serious imprecision resulting from a very small sample size and wide confidence intervals after participant dropouts related to serious adverse events (SAEs). The studies by Mähler et al., 2015 [19], Rust et al., 2021 [20], and Petracca et al., 2021 [21] were low-quality due to serious imprecision for similar reasons in contrast. Ghiasian et al., 2021 [22] was high-quality, supported by adequate sample size with power calculations, consistency of findings across outcomes, clinical relevance and trial registration. The remaining trials were judged to provide moderate-quality evidence. While generally well conducted, these studies were downgraded due to residual concerns regarding imprecision and heterogeneity, particularly the lack of power calculations, modest sample sizes, and variability in intervention characteristics and outcome measures. Across the evidence base, heterogeneity was evident both clinically and methodologically, precluding quantitative synthesis and limiting the generalisability of findings. Overall, publication bias was considered minimal, as few trials reported statistically significant or strongly positive findings. Most were registered and published in established journals.

EDSS, ARR, MRI findings, and safety/tolerability were selected to comprehensively assess the clinical efficacy and safety of polyphenol supplementation in multiple sclerosis, aligning with the objectives to evaluate patient-reported symptoms, biochemical and structural changes, and potential subgroup variability.

### 3.1. Expanded Disability Status Scale (EDSS)

Ten trials assessed the impact of polyphenol interventions on EDSS (Table 3). Four investigated EGCG at varying doses, durations, and MS subtypes—Louvera et al., 2015 [18], Mähler et al., 2015 [19], Rust et al., 2021 [20], and Bellmann-Strobl et al., 2021 [23]—but none showed statistically significant effects. Louvera et al., 2015 [18] suggested sex-specific responses, with worsening scores in women following intervention, though overall there is no evidence supporting EGCG efficacy in improving EDSS.

Three trials—Dolati et al., 2018 [24], Dolati et al., 2019 [25], and Dolati et al., 2018 [26]—consistently reported significant improvements with nanocurcumin (80 mg/day for 6 months) in RRMS, with treatment groups showing lower EDSS scores than placebo (*p* = 0.039–0.041). Interventions of cranberry extract by Gallien et al., 2014 [27] and BCM-95 curcumin by Petracca et al., 2021 [21] produced no significant EDSS changes. With SM 420 mg/day for six months, Abbasirad et al., 2021 [28] stabilised EDSS in the treatment group while it slightly worsened in placebo, though results were not statistically significant (*p* = 0.197). A smaller dose (140 mg/day for six months) reduced mean EDSS from 1.39 +/− 0.82 to 0.98 +/− 0.97 Ghiasian et al., 2021 [22], but significance could not be determined due to unreported *p*-values [35].

### 3.2. ARR (Annualised Relapse Rate)

The ARR is the average number of relapses per participant per year. Across three separate studies with different polyphenols interventions, none demonstrated a significant reduction in relapse rates compared to placebo. This was regardless of MS subtype or study duration.

An 800 mg capsule of sunphenon (>90% EGCG) compared to placebo demonstrated similar rates of relapse at around 1 every 2 years (*p* = 0.954). ARR in the intervention group was 0.47 (0.73) vs. 0.50 (0.76) in placebo, according to Bellmann-Strobl et al., 2021 [23]. Escalating doses of EGCG slightly lowered ARR, with around 1 relapse every 4–5 years but with no significant difference (*p* = 0.513). ARR in the EGCG group was 0.24 (±0.46) and in placebo was 0.19 (±0.44) in Rust et al., 2021 [20].

BCM-95 curcumin again showed no significant improvement in ARR (*p* = 0.323) at 12 months of intervention. Relapses occurred at a rate of 1–1.7 per year, demonstrating more active disease in this study population, according to Petracca et al., 2021 [21].

### 3.3. MRI Outcome

Three trials assessed radiological outcomes; none showed significant improvements in MRI measurements following polyphenol supplementation. In RRMS, an RCT comparing 800 mg/day Sunphenon (>90% EGCG) with glatiramer acetate (GA) to placebo with GA found a higher proportion without new T2w lesions in the intervention group (29% vs. 25%), though not significant. Mean new T2w lesion counts were also higher in the treatment arm (3.1 ± 6.2 vs. 1.9 ± 5.1; *p* = 0.607). No differences were identified in lesion volume, T1w hypointense lesions, contrast-enhancing lesions, or brain volume change (all *p* > 0.3) according to Bellmann-Strobl et al., 2021 [23].

In an 80-patient cohort with any MS subtype, BCM-95 Curcumin plus interferon-beta (IFN) was compared to IFN plus placebo over 12 months. Proportions without new T2w lesions were similar (20% vs. 17.5%, *p* = 0.962), with no differences in mean T2w lesion counts (0.52 ± 1.28 vs. 0.48 ± 1.87, *p* = 0.931) or mean T1 Gd-enhancing lesions (0.19 ± 0.79 vs. 0.46 ± 1.61, *p* = 0.434) according to Petracca et al., 2021 [21].

A 6–12-month study of 38 patients with PPMS/SPMS compared escalating EGCG doses to placebo. The EGCG group demonstrated a lower mean number of new T2-weighted (T2w) lesions compared with placebo (1.52 ± 4.23 vs. 3.78 ± 4.88); however, this was not significantly different (*p* = 0.146), indicating no clear evidence of reduced inflammatory lesion formation. The large standard deviations in both groups suggest substantial inter-individual variability, raising the possibility that certain patient subgroups may have responded to ECGC treatment differently. In contrast, the total T2 lesions volume was significantly larger (1040 mm^3^ vs. 520 mm^3^; *p* = 0.043) in the EGCG group, indicating that while fewer lesions may have formed, existing lesions may have enlarged or there is ongoing tissue damage. No significant differences were found in new T1w hypointense lesions, which are more closely associated with irreversible axonal injury, nor in percentage brain volume change (PBVC) or brain parenchymal fraction, suggesting that EGCG did not have a measurable effect on brain atrophy or neurodegeneration over the study period [20].

### 3.4. Safety Outcomes and Liver Function Tests (LFTs)

Seven studies reported safety outcomes and/or LFT measurements, covering five interventions at varying doses and durations (6 weeks–18 months) (*n*: 3–171). Common adverse events (AEs) were gastrointestinal symptoms, urinary tract symptoms, and upper respiratory tract infections. Given that continuation of the intervention was possible with these AEs, they are considered mild. Gallien et al., 2014 [27] aimed to reduce the frequency of UTIs in those with MS using 36 mg proanthocyanidins. There was no difference in time to the first symptomatic UTI (*p* = 0.97).

Four RCTs evaluated the AEs between intervention and placebo (Table 4). In both Petracca et al., 2021 [21] and Bellmann-Strobl et al., 2021 [23], AE rates were similar across groups, though no *p*-values were provided. Gallien et al., 2014 [27] also showed no significant differences. By contrast, Louvera et al., 2015 [18] reported a significantly higher AE rate in the intervention arm.

Three trials assessed LFTs. SM at a dose of 140 mg in Ghiasian et al., 2021 [22] showed a significant reduction in aspartate aminotransferase (*p* = 0.014) but not alanine aminotransferase (*p* = 0.35). A higher dose of 420 mg Silymarin in Abbasirad et al., 2021 [28] significantly lowered LFTs, though specific enzymes are not described. Polyphenon E significantly worsened LFTs, leading to trial termination, with incidences of 83% vs. 20% in intervention and placebo (*p* < 0.05) according to Louvera et al., 2015 [18].

Two studies reported SAEs. In Petracca et al., 2021 [21], 2.6% of the intervention developed an SAE versus none in placebo, whilst Bellmann-Strobl et al., 2021 [23] reported 10% vs. 13%, respectively. Both differences were non-significant.

## 4. Discussion

This body of evidence demonstrates limited and inconsistent clinical benefit of polyphenols, with only nanocurcumin showing statistically significant improvements in EDSS in RRMS patients. Other polyphenols including EGCG, SM cranberry extract, and BCM-95 curcumin showed no consistent effect on EDSS, ARR, or MRI outcomes across MS subtypes. Other polyphenols reviewed here may still provide supportive clinical benefits, including hepatoprotective effects and improved liver function parameters, which could be valuable in patients receiving existing disease-modifying therapies. This is one of the most comprehensive reviews to date, synthesising 13 clinical trials involving 785 participants across multiple countries and MS subtypes. This review directly compares a broad range of polyphenols across key clinical and biochemical outcome measures. The inclusion of safety and liver function markers also provides novel insights into the risk–benefit profile of long-term polyphenol use in MS.

Multiple sclerosis is driven by two interconnected processes: immune mediated neuroinflammation through blood–brain (BBB) barrier disruption and the infiltration of Th1 and Th17 lymphocytes alongside the overproduction of cytokines including IFN-γ, TNF-α, IL-1β, IL-6, and IL-17, alongside oxidative stress-derived neurodegeneration through reactive oxygen species (ROS) and reactive nitrogen species (RNS) production, mitochondrial dysfunction, and oligodendrocyte apoptosis [30].

Polyphenols act by modulating immune responses, redox balance, and BBB integrity. Polyphenols, particularly curcumin and resveratrol, have been shown to scavenge ROS and RNS, which protect oligodendrocytes and neurons. Polyphenols have been shown to reduce levels of Th1 cytokines and inhibit IL-17 production along with increasing anti-inflammatory cytokines [36]. This can reduce the autoimmune attack on myelin, again protecting neurons. Many of these mechanistic pathways have been demonstrated in Peripheral Blood Mononuclear Cells (PBMCs). Possible differences in results seen above may be due to how polyphenols are metabolised in the gut alongside gut modifications and complex pathway interactions, acting on different molecular targets and dosing differences [37].

### 4.1. EDSS

Curcumin is a natural antioxidant with anti-inflammatory and potential anticancer properties [38]. However, it is limited by poor bioavailability. Nanocarrier delivery systems [39] enhance absorption by encapsulating curcumin. In MS, curcumin may exert benefits by scavenging ROS and modulating the immune response—upregulating regulatory T-cells and suppressing pro-inflammatory cytokines like TNF-a, IL-6, and IL-1b.

EDSS is a widely used, standardised, validated tool to assess disability and progression of MS, rated from 0 (normal) to 10 (death due to MS). This tool is helpful when comparing clinical trials but has notable limitations—up to 17% inter-rater variability and underrepresentation of cognitive and upper limb functions [40]. EDSS is disproportionately weighted towards mobility and less sensitive to cerebral pathology, including fine motor control, fatigue, and cognitive processing speed, symptoms that contribute substantially to disease burden. Hence, the absence of EDSS improvement in these studies does not necessarily indicate a lack of clinical effect, just that the right tool to measure these may not have been utilised. These factors should be considered when interpreting the findings, as no studies provided detailed protocols for EDSS assessment. The absence of harmonised outcome measures across trials represents a major barrier to evidence synthesis and may partly explain the inconsistent findings observed. A forest plot to summarise the results was not possible due to the lack of *p*-values and risk/odds ratios. The incorporation of multidimensional tools, such as the Multiple Sclerosis Functional Composite (MSFC), alongside cognitive testing and fatigue-specific instruments, would provide a more comprehensive assessment of disease status and therapeutic response [41]. Results with SM are harder to interpret. SM has shown promise in reducing demyelination by suppressing inflammatory cytokines and restoring ApoE in animal models [42]. Differences in outcomes from these human trials may be due to cytokine pathway saturation in higher doses of SM or could become pro-oxidant at higher levels, this being theoretical.

### 4.2. ARR

ARR is influenced by MS subtype, concurrent infections, disease duration, existing treatment regimens, age, and sex, among others [43]. This may explain the lack of significant results, but without subgroup analysis, the true intervention impact is unclear.

### 4.3. MRI

There is little evidence supporting polyphenol supplementation in modifying radiological markers of MS disease activity or neurodegeneration. Brain volume measures like PBVC and brain parenchymal fraction remained unchanged; this may reflect insufficient follow-up duration, small sample sizes, or limited neuroprotective effects of polyphenols. Given that average brain volume loss per year in those with MS is 0.5% per year versus 0.1% in healthy populations, results may stem from minimal changes being seen across the study duration time [44]. An intriguing finding was that while the number of new T2 lesions did not differ between EGCG and placebo, lesion volume was larger with EGCG after 12 months—though its clinical significance is unclear. T2 lesion count, which is part of the 2017 McDonald criteria, correlates weakly to moderately with disability [45], whereas lesion volume better reflects disease burden and correlates with EDSS scores [40]. In the study performed by Rust et al., 2021 [20], EDSS scores were assessed alongside T2 lesion number. Notably, EDSS scores worsened in both the intervention and placebo groups, with no significant difference at trial completion. This suggests that, although lesion volume increased in the intervention group, the clinical significance of this is limited.

### 4.4. Safety Outcomes

SM has demonstrated hepatoprotective effects, with two trials reporting improved LFTs, likely due to its antioxidant properties—scavenging free radicals, preventing lipid peroxidation, and stabilising hepatocyte membranes [46]. Additionally, SM enhances the liver’s detoxifying capacity by stimulating ribosomal RNA polymerase I activity, which improves protein synthesis and hepatocyte regeneration [47]. This may also promote more efficient metabolism of SM, reducing risk of toxic metabolite accumulation. Clinically, liver enzyme elevations may necessitate increased monitoring, meaning more frequent blood tests, treatment interruption or discontinuation, particularly in patients receiving hepatically metabolised disease-modifying therapies. Therefore, any additional intervention that can reduce the risk of hepatotoxicity or adverse events of existing MS treatments may prove useful. Hence, this is considered a more serious adverse event. An outlier study was Lovera et al., 2015 [18], reporting significantly more AEs, though unclear if dose- or duration-dependent. In contrast, the green tea extract sunphenon (>90% EGCG) used in Bellmann-Strobl et al., 2021 [23] showed no difference in AEs compared to placebo, despite a longer study duration and identical total daily dosing. Tolerability differences may stem from bioavailability, dietary factors, other drug interactions, or dosing frequency [45,46,47,48,49]. Twice-daily dosing could affect metabolism depending on EGCG half-life [50]. Future trials should compare EGCG doses and formulations to better assess safety. Given that most AEs were mild, including those of nausea, respiratory, or urinary symptoms, the marked difference is not of significant concern.

Similar polyphenolic compounds reviewed in this paper have been shown to improve disease activity scores and reduce inflammatory and oxidative stress markers in rheumatoid arthritis (RA), which is also an autoimmune disorder. Curcumin was among the most extensively studied compounds in this context. Although the administration regimens, duration of polyphenol supplementation and underlying pathophysiology differs from that observed in MS, several common mechanistic pathways are affected in both conditions. These include inhibition of NF-κB, modulation of pro-inflammatory cytokines such as TNF-α and IL-6, and attenuation of oxidative stress. Notably, Long et al. [51] emphasised the need for additional RCTs to determine the optimal efficacy of polyphenol supplementation for achieving meaningful clinical and biochemical benefits, a conclusion that closely aligns with the findings of this present review.

Findings here mirror that in Alzheimer’s disease there was no significant EDSS improvement with EGCG—a systematic review of green tea found mixed effects on cognition. This same systematic review identified no significant improvement with curcumin at doses 1–4 g, these differences may be due to poor bioavailability. Studies in this review utilised nanocarriers which may explain differences in results [52]. Nanocurcumin is the only polyphenol examined here which utilised nanocarriers. This may have avoided the altered metabolism and absorption which can be seen in those which did not use nanocarriers, possibly owing to their more impactful findings. Other polyphenols may have been limited by poor oral bioavailability due to low aqueous solubility, rapid intestinal metabolism, and extensive first-pass hepatic degradation, resulting in low systemic and central nervous system concentrations. Encapsulation on curcumin improves plasma concentration and prolongs circulation time [53,54]. This may allow nanocurcumin to exert more consistent antioxidant and anti-inflammatory effects. The enhanced bioavailability of nanocurcumin may explain the observed improvements in EDSS compared with other polyphenols. However, the absence of subgroup analysis within the RCTs reviewed here makes it difficult to determine whether these effects are attributable to the intrinsic properties of the polyphenol itself or to the improved bioavailability conferred by the nanocarrier formulation.

The findings highlight several critical gaps in the current evidence base. Clinical outcomes can be influenced by patient demographics, concomitant therapies, and concurrent infections. Future studies must therefore incorporate subgroup analyses and intention-to-treat approaches to disentangle these confounding factors and determine the true impact of polyphenol supplementation. Significant heterogeneity among studies precluded the conduct of meta-analysis as many trials failed to provide sufficient statistical information including *p*-values and confidence intervals [55]. To advance the field, larger and well-designed multicentre RCTs are needed, guided by robust power calculations. Such trials should stratify participants by relevant variables to clarify the true impact of polyphenols.

The safety findings of this review may imply that polyphenols should be considered adjunctive, to reduce adverse events of existing MS therapies. Or, if used along, baseline liver function assessment and periodic monitoring should be recommended. Predefined liver enzyme thresholds for dose adjustment or discontinuation and consistent reporting of adverse event severity should be used.

Future research should also explore dosing strategies more systematically. Trials that directly compare varying doses, durations and delivery systems could identify optimal regimens for therapeutic benefit while maintaining safety.

### 4.5. Limitations

This review has several limitations that should be acknowledged. First, study screening was conducted by a single reviewer, which may increase the risk of selection bias. To mitigate this risk, the screening process was guided by clearly predefined inclusion criteria and minimal exclusion criteria. In addition, a second reviewer independently verified the extracted data, providing an additional layer of quality control and reducing the likelihood of data extraction errors.

Second, the review was restricted to English-language publications and employed a date-limited search strategy focused on recent practice. These pragmatic decisions were made to ensure feasibility and relevance to contemporary clinical and healthcare contexts. While the inclusion of non-English studies may have provided additional perspectives, this was not feasible given the small review team. Similarly, inclusion of earlier trials may have complicated data synthesis due to the use of heterogeneous and outdated outcome measures. Given that polyphenol research is a rapidly evolving field, it is unlikely that older studies would have substantially contributed to the identification of clinically meaningful outcomes beyond those captured in more recent trials.

The substantial heterogeneity observed across the included studies reflects the multiple interacting clinical and methodological factors. Trials included enrolled heterogenous MS populations with 6 of the 13 included studies not stating the distribution of MS subtypes among participants. Although MS subtypes share overlapping pathophysiological mechanisms, they differ in inflammatory activity, disease trajectory and rates of progression which may influence response to polyphenol supplementation. Baseline disability also varied across studies, clinically meaningful changes in outcome measures such as EDSS may not have manifested within the follow up period employed. The concomitant use of disease-modifying therapies that may interact with polyphenols may confound or mask the effect of polyphenols. Intervention-related variability was also pronounced, including differences in polyphenol class, dose, duration, formulation, and delivery system, all of which critically influence bioavailability and central nervous system penetration. Notably, although nanocurcumin demonstrated greater improvements in EDSS compared with other polyphenols, the absence of subgroup analysis makes it difficult to ascertain if the findings were a result of the polyphenol itself or the enhanced bioavailability. This highlights an important area for the development of nanocarrier-based approaches for other polyphenols to improve their bioavailability. Sources of heterogeneity are summarised in Supplementary Table S1.

## 5. Conclusions

Most notably, nanocurcumin may improve disability outcomes in those with RRMS, as measured by EDSS in MS. Other polyphenols including EGCG, SM, cranberry extract, and BCM-95 show no consistent benefit to disability, relapse rate, or MRI markers of disease activity. Most polyphenols exhibit favourable safety profiles with limited evidence demonstrated here of any increased risk of AE/SAE and with well tolerated side effects. Overall, while the evidence base remains limited and heterogeneous, this synthesis suggests that nanocurcumin holds promise as a complementary strategy for MS management. However, robust, large-scale, and methodologically rigorous trials are urgently needed to validate these preliminary findings and to determine the optimal formulations and dosing regimens that could translate into meaningful clinical benefits.

## Figures and Tables

**Table 3:** Change in EDSS scores from baseline to study completion for intervention and placebo groups across 10 RCTs of polyphenol supplementation in MS.

Study	Intervention	EDSS Change (Intervention)	EDSS Change (Placebo)	*p*-Value
Louvera et al., 2015 [18]	400 mg EGCG BD	No data—no significant difference	No data—no significant difference	Not given
Mähler et al., 2015 [19]	300 mg EGCG BD	Men: 0/Women: 0.3	Men: 0/Women: −0.2	Not given
Rust et al., 2021 [20]	100–1200 mg EGCG (up-titrating)	0.26 ± 0.45	0.57 ± 0.99	0.421
Petracca et al., 2021 [21]	BCM-95 micronised curcumin with turmeric essential oils (500 mg orally)	No data	No data	0.176
Ghiasian et al., 2021 [22]	140 mg silymarin OD	0.41	0.04	Not given
Dolati et al., 2018 [24]	80 mg nanocurcumin/day	0.98 ± 0.29	1.72 ± 1.06	0.03
Dolati et al., 2019 [25]	80 mg nanocurcumin/day	0.64	0.14	0.041
Dolati et al., 2018 [26]	80 mg nanocurcumin/day	No data	No data	0.041 (no data given)
Gallien et al., 2014 [27]	Oral cranberry extract (18 mg proanthocyanidins) BD	No data—no significant difference	No data—no significant difference	Not given
Abbasirad et al., 2021 [28]	SM (420 mg in three divided doses)	0	0.12	0.197

**Table 4:** Percentage of patients experiencing AEs during four trials using different polyphenols, comparing intervention and placebo.

Study [Reference]	Intervention	Percentage of Patients with AEs in Intervention	Percentage of Patients with AEs in Placebo	*p*
Louvera et al., 2015 [18]	EGCG 400 mg BD for 12 months	83%	20%	<0.05
Petracca et al., 2021 [21]	BCM-95 Curcumin for 12 months	42.1%	40%	
Bellmann-Strobl et al., 2021 [23]	800 mg capsule of sunphenon (GTE containing >90% EGCG) for 18 months	97%	97%	
Gallien et al., 2014 [27]	Oral cranberry extract with 18 mg proanthocyanidins BD for 12 months	17.1	20.5	0.32

## Data Availability

No new data were created or analyzed in this study. Data sharing is not applicable to this article.

## Supplementary Materials

The following supporting information can be downloaded at: https://www.mdpi.com/article/10.3390/nu18121875/s1, Table S1: Areas for heterogeneity within this systematic review.
