# Supplementary material for: The Effect of Polyphenol Supplementation in People with Multiple Sclerosis: A Systematic Review of Clinical Trials
Source: Nutrients. 2026 Jun 10;18(12):1875. doi: 10.3390/nu18121875 (PMC13304819; doi:10.3390/nu18121875)
Supplement: Supplementary file 1 [file nutrients-18-01875-s001.zip › nutrients-4252568-supplementary.pdf]

Source of heterogeneity	Number of populations sampled	Level of heterogeneity	Explanation
Subtypes of MS	3	High	Different subtypes of MS, although have overlapping pathophysiology, subtle differences may alter effect of polyphenols.
Country	6	Moderate	Geographical variation introduces differences in diet, genetics, healthcare systems and diagnostic criteria.
Age of participants	All studies within 23-59	Low	age can influence disease progression and metabolism; the limited spread reduces its impact as a major source of inconsistency.
Year of study	2009-2024	Low	MS management has not greatly advanced in this time period.
Trial duration	2-12 months	Moderate- most studies were conducted for 6 months	Variation in duration of study can lead to discrepancies in whether effects are observed.
Intervention	11- varying doses and polyphenol subtype	High	Intervention is one of the highest areas of heterogeneity with different polyphenols having distinct mechanisms of action.
Sample size	10-122	High	Small studies are underpowered and more prone to random error and exaggerated effect sizes. Larger studies provide more reliable estimates. This imbalance increases variability and reduces comparability across studies.
Concomitant disease modifying therapies	3 (stable on therapy, no concomitant therapy, concomitant fingolimod)	High	Concomitant disease modifying therapies may themselves interact with polyphenols and this effect is unknown.
Baseline disability	4 (Mild, moderate, high and concomitant fatigue)	Moderate	Often difference in disability was an outcome measure.
Outcome assessment	11 (MRI, EDSS, safety, energy expenditure, fat oxidation, functional measures, biomarkers, oxidative stress markers, miRNA expression, immunological markers, liver function tests)	High	Broad range of outcome assessment was used with lack of standardisation for many measures limits comparison.

Supplementary table S1

The above table describes areas for heterogeneity within this systematic review.
